# Blocks in Tricarboxylic Acid Cycle of Salmonella enterica Cause Global Perturbation of Carbon Storage, Motility, and Host-Pathogen Interaction

**DOI:** 10.1128/mSphere.00796-19

**Published:** 2019-12-11

**Authors:** Janina Noster, Nicole Hansmeier, Marcus Persicke, Tzu-Chiao Chao, Rainer Kurre, Jasmin Popp, Viktoria Liss, Tatjana Reuter, Michael Hensel

**Affiliations:** a Abt. Mikrobiologie, Universität Osnabrück, Osnabrück, Germany; b Department of Biology, Luther College at University of Regina, Regina, Canada; c Microbial Genomics and Biotechnology, Center for Biotechnology, Universität Bielefeld, Bielefeld, Germany; d Institute of Environmental Change & Society, University of Regina, Regina, Canada; e Integrated Bioimaging Facility iBiOs, Universität Osnabrück, Osnabrück, Germany; f Center of Cellular Nanoanalytics Osnabrück, Universität Osnabrück, Osnabrück, Germany; University of Iowa

**Keywords:** TCA cycle, glycogen metabolism, chemotaxis, phagocytosis

## Abstract

The tricarboxylic acid (TCA) cycle is a central metabolic hub in most cells. Virulence functions of bacterial pathogens such as facultative intracellular Salmonella enterica serovar Typhimurium (*S*. Typhimurium) are closely connected to cellular metabolism. During systematic analyses of mutant strains with defects in the TCA cycle, a strain deficient in all fumarase isoforms (Δ*fumABC*) elicited a unique metabolic profile. Alongside fumarate, *S*. Typhimurium Δ*fumABC* accumulates intermediates of the glycolysis and pentose phosphate pathway. Analyses by metabolomics and proteomics revealed that fumarate accumulation redirects carbon fluxes toward glycogen synthesis due to high (p)ppGpp levels. In addition, we observed reduced abundance of CheY, leading to altered motility and increased phagocytosis of *S*. Typhimurium by macrophages. Deletion of glycogen synthase restored normal carbon fluxes and phagocytosis and partially restored levels of CheY. We propose that utilization of accumulated fumarate as carbon source induces a status similar to exponential- to stationary-growth-phase transition by switching from preferred carbon sources to fumarate, which increases (p)ppGpp levels and thereby glycogen synthesis. Thus, we observed a new form of interplay between metabolism of *S*. Typhimurium and cellular functions and virulence.

**IMPORTANCE** We performed perturbation analyses of the tricarboxylic acid cycle of the gastrointestinal pathogen Salmonella enterica serovar Typhimurium. The defect of fumarase activity led to accumulation of fumarate but also resulted in a global alteration of carbon fluxes, leading to increased storage of glycogen. Gross alterations were observed in proteome and metabolome compositions of fumarase-deficient *Salmonella*. In turn, these changes were linked to aberrant motility patterns of the mutant strain and resulted in highly increased phagocytic uptake by macrophages. Our findings indicate that basic cellular functions and specific virulence functions in *Salmonella* critically depend on the proper function of the primary metabolism.

## Abstract (precis)

We performed perturbation analyses of the tricarboxylic acid cycle of the gastrointestinal pathogen Salmonella enterica serovar Typhimurium. The defect of fumarase activity led to accumulation of fumarate but also resulted in a global alteration of carbon fluxes, leading to increased storage of glycogen. Gross alterations were observed in proteome and metabolome compositions of fumarase-deficient *Salmonella*. In turn, these changes were linked to aberrant motility patterns of the mutant strain and resulted in highly increased phagocytic uptake by macrophages. Our findings indicate that basic cellular functions and specific virulence functions in *Salmonella* critically depend on the proper function of the primary metabolism.

## INTRODUCTION

The central carbon metabolism (CCM) is essential for all prototrophic bacteria because it provides energy, as well as precursors for biosynthesis of a large number of biomolecules. In particular, the tricarboxylic acid (TCA) cycle produces the reductive equivalents for the electron transport chain and the carbon backbone for various amino acids, making it an important hub for efficient bacterial metabolism in changing environments (1, 2). Several endogenous factors, such as the energy status of the cell, influence TCA cycle activity. For example, the activity of the isocitrate dehydrogenase is allosterically stimulated by ADP (3), whereas α-ketoglutarate dehydrogenase is inhibited by its products succinyl coenzyme A (CoA) and NADH (4). In addition, bacterial citrate synthesis is controlled by allosteric inhibition of citrate synthase by ATP and NADH (5). However, TCA cycle activity is also influenced by exogenous factors, such as exposure to antibiotics and reactive oxygen species (ROS), which target sensitive enzymes harboring Fe-S clusters (6, 7).

Salmonella enterica serovar Typhimurium (*S*. Typhimurium) is an invasive facultative intracellular pathogen, the causative agent of human gastroenteritis, and serves as a model organism for systemic *Salmonella* infections. The divergent host niches colonized during infection require *S*. Typhimurium to adapt its metabolism from the intestinal lumen, which is a nutrient-rich environment with a competing microbiome (8), to severe nutritional restrictions and ROS attacks inside the so-called *Salmonella*-containing vacuole (SCV) during intracellular life within host cells (9, 10). Its versatile and robust metabolism (11) makes *S*. Typhimurium an ideal model organism to study the interconnection of metabolism and virulence functions.

To address the role of the TCA cycle in pathometabolism of *S*. Typhimurium, we analyzed the effect of perturbations of the TCA cycle using a set of mutant strains each defective in one enzymatic step. Our previous study indicated that TCA cycle perturbations induced in *S*. Typhimurium by oxidative stress result from damage of Fe-S cluster-containing enzymes (12). Accordingly, a mutant strain deficient in all three fumarase isoforms (Δ*fumABC*) accumulated large amounts of TCA intermediate fumarate but also showed the remarkable phenotype of increased phagocytosis by murine macrophages. These observations pointed toward a link between TCA cycle metabolite fumarate and cellular functions of *S*. Typhimurium.

The C_4_-dicarboxylate fumarate recently gained increasing interest due to various links between metabolism and bacterial pathogenesis. In enterohemorrhagic Escherichia coli (EHEC), fumarate is essential for full virulence in a Caenorhabditis elegans infection model where it regulates the expression of a tryptophanase by the transcription factor Cra (13). In Mycobacterium tuberculosis, fumarase deficiency was shown to be fatal due to protein and metabolite succination (14). Other studies demonstrated fumarate as a factor that increases the frequency of persister formation or modulates motility and chemotaxis in E. coli (15–17).

In this work, we conducted metabolomics and proteomics studies to characterize the metabolic landscape of *S*. Typhimurium Δ*fumABC*. By this dual-omics approach, we elucidated a new example for the interplay between metabolism and cellular functions and virulence in *S*. Typhimurium.

## RESULTS

### Effects of TCA cycle enzyme deletion on the carbon metabolism of *S*. Typhimurium.

For a global analysis of the effects of perturbations of the TCA cycle on pathometabolism of *S*. Typhimurium, we generated a set of isogenic *S*. Typhimurium mutant strains, each defective in one reaction of the TCA cycle. Using this set of strains in comparison to *S*. Typhimurium wild type (WT), we performed metabolomics analyses of stationary cultures, grown for 18.5 h in rich medium (LB broth), and analyzed samples as described before (12). Metabolomics revealed that the Δ*fumABC* strain, deficient in all fumarase isoforms, had a highly aberrant metabolic profile distinct from that of other mutant strains. Besides a strong accumulation of fumarate (115-fold compared to WT), *S*. Typhimurium Δ*fumABC* contained significantly increased amounts of glycolysis and pentose phosphate pathway (PPP) intermediates.

**FIG 1:**
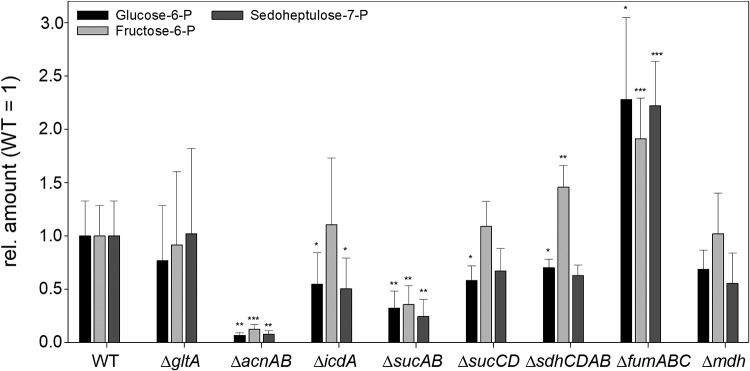
Defects in TCA cycle enzymes affect metabolite concentrations. *S*. Typhimurium WT and mutant strains defective in TCA cycle enzymes were grown aerobically in LB broth for 18.5 h at 37°C. Cells were harvested and disrupted, and metabolites were extracted for subsequent GC-MS analyses. Metabolite concentrations were normalized to WT levels, and means and standard deviations for at least four biological replicates are shown. Statistical analyses were performed by Student’s *t* test, and significances are indicated as follows: *, *P* < 0.05; **, *P *< 0.01; ***, *P* < 0.001.

Moreover, the Δ*fumABC* strain exhibited increased levels of glucose-6-phosphate (G6P), fructose-6-phosphate (F6P) and sedoheptulose-7-phosphate (S7P), whereas all other mutant strains exhibited decreased or unchanged levels compared to WT (Fig. 1; see also Table S1 in the supplemental material). This observation indicates distinct and unique impacts of the fumarase deletions on carbon flux.

10.1128/mSphere.00796-19.6 TABLE S1 Metabolomics, TCA cycle enzyme deletion strains and Δ*glgA* and Δ*fumABC* Δ*glgA* strains. Download Table S1, XLSX file, 0.3 MB. Copyright © 2019 Noster et al. 2019 Noster et al. This content is distributed under the terms of the Creative Commons Attribution 4.0 International license.

Only a mutant strain deficient in succinate dehydrogenase also showed a larger level of F6P, but not to the same extent as observed for the Δ*fumABC* mutant. Furthermore, there was a strong accumulation of aspartate, likely arising from the large pool of fumarate by the action of aspartate ammonia-lyase AspA (Table S2).

10.1128/mSphere.00796-19.7 TABLE S2 Proteomics, WT versus Δ*fumABC* strains. Download Table S2, XLSX file, 0.07 MB. Copyright © 2019 Noster et al. 2019 Noster et al. This content is distributed under the terms of the Creative Commons Attribution 4.0 International license.

In our previous analyses of ROS-induced damage of TCA cycle enzymes in *S*. Typhimurium pathometabolism, we found that a mutant strain unable to detoxify endogenously generated ROS was attenuated in intracellular proliferation. Surprisingly, this mutant strain was internalized by macrophages at higher rates than *S*. Typhimurium WT (12). Endogenous ROS cause damage of Fe-S cluster-containing TCA cycle enzymes, and also a Δ*fumABC* strain was internalized by macrophages at a 15-fold-higher rate than WT *S*. Typhimurium, without defects in intracellular proliferation. These observations point toward a link between the function of the TCA cycle and the virulence properties of *S*. Typhimurium, which prompted us to characterize the *S*. Typhimurium Δ*fumABC* strain in detail.

### Quantitative proteomic and metabolic profiling reveal alterations in the central carbon metabolism of *S*. Typhimurium Δ*fumABC*.

**FIG 2:**
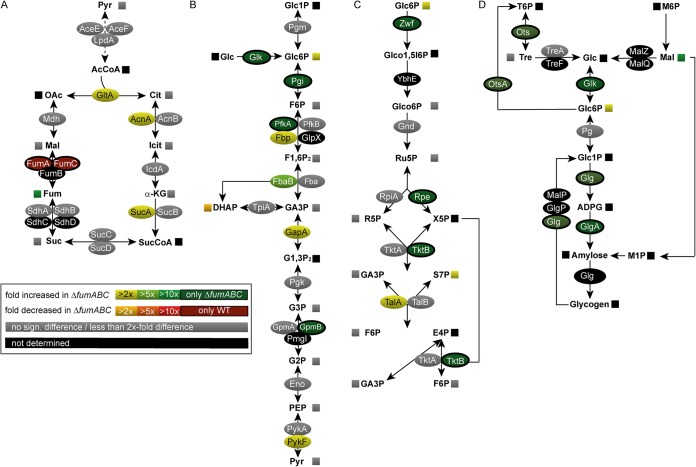
Deletion of fumarases leads to changes in carbon fluxes and amounts of metabolic enzymes. *S*. Typhimurium WT and Δ*fumABC* strains were grown aerobically in LB broth for 18.5 h at 37°C. For the proteomic approach, harvested cells were lysed and proteins were precipitated with 10% TCA. After trypsin digestion, the peptides were analyzed by quantitative LC-MS^E^ (where ^E^ stands for elevated offset). For metabolomics analyses, harvested cells were disrupted and metabolites were extracted for GC-MS analysis. Heat map colors of oval symbols indicate relative changes in amounts of enzymes detected for Δ*fumABC* mutant compared to WT. Heat map colors of square symbols indicate relative changes in amounts of metabolites determined in Δ*fumABC* mutant compared to WT. Gray symbols indicate less than 2-fold or nonsignificant differences in enzyme or metabolite amounts. Quantitative data are shown for TCA cycle (A), glycolysis (B), pentose phosphate pathway (C), and glycogen synthesis (D). Data represent means from at least four or three biological replicates for the metabolomics or proteomics analyses, respectively. Statistical analyses were performed by Student’s *t* test, and all data shown have significance differences between the two strains of *P* < 0.05 or lower.

We first performed proteomic and metabolic profiling of *S*. Typhimurium WT and Δ*fumABC* strains after culture in rich media (LB broth) for 18.5 h and analyzed samples as described previously (12). As anticipated from genotype and fumarate accumulation, fumarases were not detected in the fumarase-deficient strain. We did not detect changes in other TCA cycle intermediates (Fig. 2). However, we observed increased amounts of citrate synthase (GltA), aconitase A (AcnA), and α-ketoglutarate dehydrogenase component (SucA) by 2.05- to 2.72-fold. With respect to catabolism of hexoses, and in line with higher concentrations of G6P (2.28-fold) and slight increment of F6P (1.91-fold), increased amounts of the corresponding enzymes were detected in the Δ*fumABC* strain. Glucokinase (Glk), glucose-6-phosphate isomerase (Pgi), phosphofructokinase A (PfkA), and phosphoglycerate mutase (GpmB) were identified only in the Δ*fumABC* strain, and we determined 2.27- to 5.42-fold-increased amounts of fructose-1,6-bisphosphatase class 1 (Fbp), fructose-bisphosphate aldolase B (FbaB), glyceraldehyde-3-phosphate dehydrogenase (GapA), and pyruvate kinase I (PykF). The increased amount of S7P can be correlated with larger amounts of glucose-6-phosphate dehydrogenase (Zwf), ribulose-phosphate-3-epimerase (Rpe), and transketolase B (TktB), detected only in the proteome of the Δ*fumABC* strain. Furthermore, transaldolase A (TalA) was increased 3.29-fold.

In addition, we observed only in the proteome of *S*. Typhimurium Δ*fumABC* key enzymes of glycogen biosynthesis, i.e., glycogen synthase (GlgA), glucose-1-phosphate adenylyltransferase (GlgC), glycogen debranching enzyme (GlgX), and trehalose-phosphate synthase (OtsA), as well as trehalose-phosphate phosphatase (OtsB) (Fig. 2D). Together with the detected accumulation of maltose (10-fold) and trehalose (2-fold), these data suggest an increased glycogen accumulation in *S*. Typhimurium Δ*fumABC* compared to the WT.

**FIG 3:**
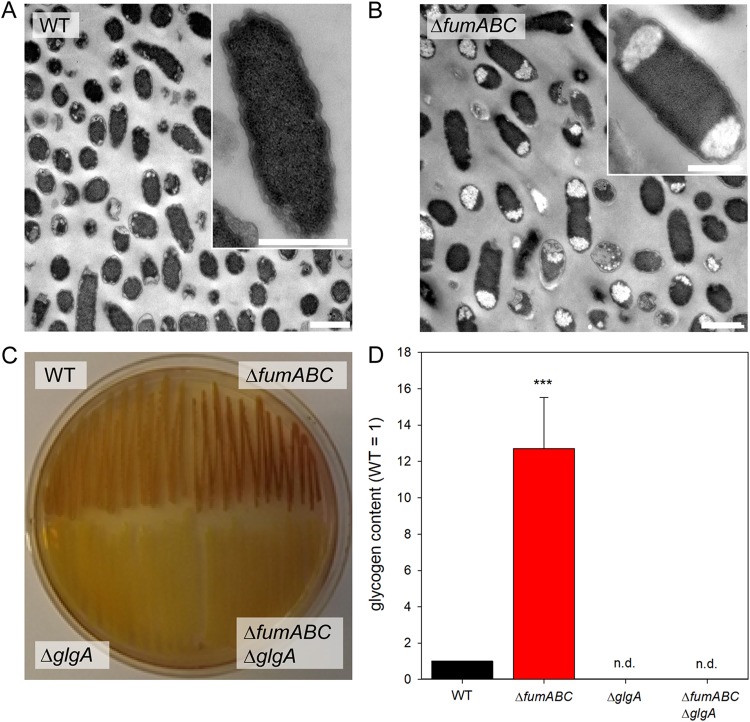
Deletion of fumarases leads to increased glycogen accumulation. (A and B) *S*. Typhimurium WT (A) and Δ*fumABC* (B) strains were grown aerobically for 18.5 h at 37°C in LB broth. Cells were fixed, dehydrated, and resin embedded, and ultrathin sections were prepared for TEM. Massive accumulations of polymers in the polar regions of Δ*fumABC* cells were observed frequently. Bars, 1 μm (overview), 500 nm (detail). (C) *S*. Typhimurium WT, Δ*glgA*, Δ*fumABC*, and Δ*fumABC* Δ*glgA* strains were grown on LB agar plates for 18.5 h at 37°C. Potassium iodine staining was performed, and brownish color indicates intercalation of iodine with glycogen. (D) Quantification of glycogen contents of *S*. Typhimurium strains grown aerobically for 18.5 h in LB broth. Glycogen was degraded to glucose monomers using amyloglucosidase, and resulting glucose was phosphorylated to G6P. G6P was oxidized by G6P dehydrogenase in the presence of NAD, being reduced to NADH. Glucose concentrations were proportional to OD_340_. By subtraction of free glucose concentrations (sample without amyloglucosidase) from total glucose concentrations, glycogen amounts were quantified. Glycogen concentrations were normalized to WT (=1), and error bars represent standard deviations from four biological replicates. n.d., not detected. Statistical analyses were performed by Student’s *t* test, and significances are indicated as follows: ***, *P* < 0.001.

To test for increased glycogen storage, bacterial cultures grown on LB agar were treated with potassium iodine for glycogen staining (18). While *S*. Typhimurium WT was only lightly stained, the intense brown color of *S*. Typhimurium Δ*fumABC* colonies indicated high accumulation of glycogen (Fig. 3C). We next applied transmission electron microscopy (TEM) of ultrathin sections of *S*. Typhimurium WT (Fig. 3A) and Δ*fumABC* cells (Fig. 3B). Granular aggregates of low electron density were observed in the polar regions of *S*. Typhimurium Δ*fumABC*, but to a far lesser extent in WT cells. Accordingly, enzymatic quantification revealed 12-fold-increased glycogen content in *S*. Typhimurium Δ*fumABC* compared to WT (Fig. 3D). Complementation of *S*. Typhimurium Δ*fumABC* by plasmids harboring *fumAC* or *fumB* genes restored WT levels of glycogen (see Fig. S1A in the supplemental material). These data indicate that fumarate accumulation in *S*. Typhimurium Δ*fumABC* is a key factor for biasing the glycogen metabolism toward altered carbon fluxes and increased glycogen storage.

10.1128/mSphere.00796-19.1 FIG S1 Glycogen accumulation in the Δ*fumABC* mutant strain is dependent on the deletion of all fumarase isoforms and an intact glycogen synthase. Quantification of glycogen contents of *S*. Typhimurium strains occurred as described in the Fig. 3 legend. (A) Complementation of the Δ*fumABC* deletion strain harboring plasmids carrying the intact genes *fumAC* or the *fumB* gene under the control of their native promoter or the empty vector, respectively. (B) Complementation of the Δ*fumABC* Δ*glgA* deletion strain with plasmids carrying the intact gene *glgA* or the empty vector, respectively. Glycogen concentrations were normalized to WT (=1), and error bars represent standard deviations from two independent biological replicates, each consisting of two technical replicates. Download FIG S1, TIF file, 5.4 MB. Copyright © 2019 Noster et al. 2019 Noster et al. This content is distributed under the terms of the Creative Commons Attribution 4.0 International license.

### Deletion of glycogen synthase GlgA decreases amounts of G6P, F6P, and S7P in *Salmonella* WT and Δ*fumABC* strains.

To further investigate the connection of glycogen biosynthesis and fumarate accumulation, we blocked glycogen synthesis by deletion of *glgA*, which encodes the glycogen synthase, in the Δ*fumABC* mutant, resulting in the *S*. Typhimurium Δ*fumABC* Δ*glgA* double mutant. We verified the loss of glycogen production in the *glgA*-deficient strain with potassium iodine staining (Fig. 3C) and TEM analyses (Fig. S2) as before and were able to restore the original phenotype by complementation with a plasmid harboring *glgA* (Fig. S1B).

10.1128/mSphere.00796-19.2 FIG S2 Absence of granular accumulation in *S*. Typhimurium Δ*fumABC* by glycogen synthase deletion. Electron micrographs of *S*. Typhimurium Δ*fumABC* Δ*glgA*, aerobically grown for 18.5 h in LB broth. Note the absence of polymer accumulations in the polar regions of the bacterium. Bars, 1 μm (overview), 0.5 μm (detail). Download FIG S2, TIF file, 0.6 MB. Copyright © 2019 Noster et al. 2019 Noster et al. This content is distributed under the terms of the Creative Commons Attribution 4.0 International license.

**FIG 4:**
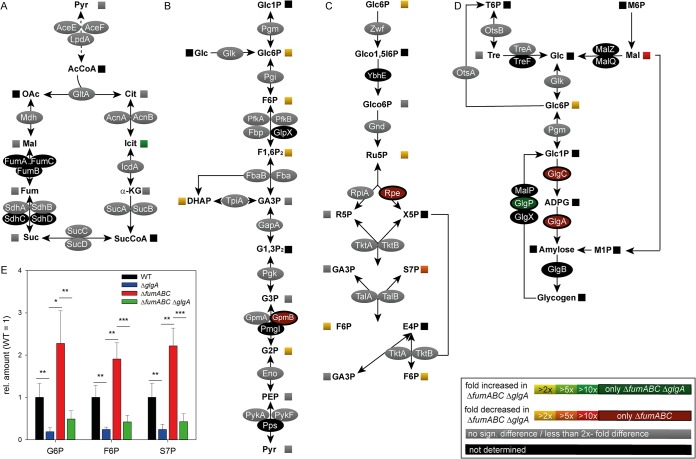
Deletion of glycogen synthase in a Δ*fumABC* strain restores carbon fluxes without changes in amounts of metabolic enzymes. (A to D) *S*. Typhimurium Δ*fumABC* and Δ*fumABC* Δ*glgA* strains were grown aerobically in LB broth for 18.5 h at 37°C. Analyses of metabolic enzymes and metabolites were performed as described for Fig. 2, and comparisons of *S*. Typhimurium Δ*fumABC* Δ*glgA* to the Δ*fumABC* mutant are shown for TCA cycle (A), glycolysis (B), pentose phosphate pathway (C), and glycogen synthesis (D). Data represent means from at least four or three biological replicates for the metabolomics or proteomics analyses, respectively. (E) The concentrations of metabolites glucose-6-phosphate (G6P), fructose-6-phosphate (F6P), and sedoheptulose-7-phosphate (S7P) were determined and normalized to WT (=1). Statistical analyses were performed by Student’s *t* test, and all data shown have significance differences between the two strains of *P < *0.05 or lower: *, *P* < 0.05; **, *P* < 0.01; ***, *P* < 0.001.

Subsequently, we performed quantitative comparative proteomics and metabolomics of *S*. Typhimurium Δ*fumABC* Δ*glgA* and compared the obtained profiles with those of *S*. Typhimurium Δ*fumABC* (Fig. 4 and Tables S1 and S3). Deletion of glycogen synthase did not affect amounts of metabolic enzymes in glycolysis, PPP, and TCA cycle but decreased the abundance of glucose-1-phosphate adenylyltransferase GlgC, an enzyme catalyzing the synthesis of ADP-d-glucose (ADPG). Metabolite analyses by gas chromatography-mass spectrometry (GC-MS) revealed strong decrease of G6P, F6P, and S7P if *glgA* is deleted (Fig. 4E). Furthermore, the amount of trehalose was increased by 30%, while amounts of maltose were 100-fold reduced in *S*. Typhimurium Δ*fumABC* Δ*glgA*.

10.1128/mSphere.00796-19.8 TABLE S3 Proteomics, Δ*fumABC* versus Δ*fumABC* Δ*glgA* strains. Download Table S3, XLSX file, 0.07 MB. Copyright © 2019 Noster et al. 2019 Noster et al. This content is distributed under the terms of the Creative Commons Attribution 4.0 International license.

We conclude that altered fluxes through glycolysis and PPP in a fumarase-deficient strain are induced by increased glycogen synthesis. Abrogation of storage compound synthesis by *glgA* knockout normalized metabolite levels, due to modified enzyme activities and regulative mechanisms, rather than altered protein amounts.

### Fumarate-induced stringent response influences *Salmonella* physiology.

**FIG 5:**
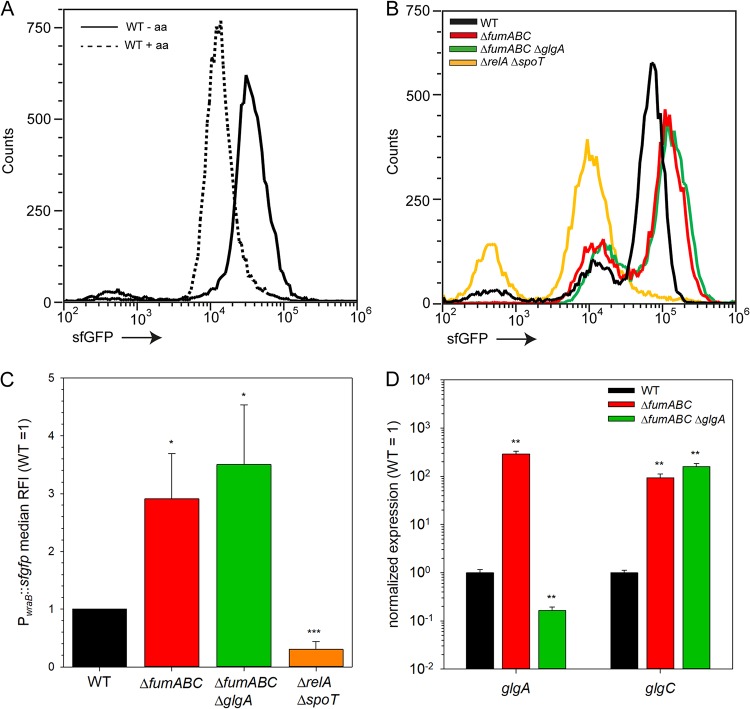
Differential expression of glycogen-synthesizing enzymes due to increased (p)ppGpp levels in Δ*fumABC* strain. (A) *S*. Typhimurium WT harboring a dual-color fluorescence reporter for *wraB* was cultured in LB o/n and subcultured in minimal medium with or without amino acid (aa) supplementation (dashed or solid line, respectively). After 3 h of growth, cells were subjected to flow cytometry and sfGFP fluorescence intensity (BL1-H) was recorded. Shown is one representative of three independent biological replicates. (B) Representative data from WT, Δ*fumABC*, Δ*fumABC* Δ*glgA*, and Δ*relA* Δ*spoT* strains harboring the *wraB* reporter grown o/n in LB broth. (C) Medians of relative sfGFP fluorescence intensities (RFI) of strains shown in panel B. Data were normalized to WT (=1) and represent average values and standard deviations from three biological replicates. (D) WT, Δ*fumABC*, and Δ*fumABC* Δ*glgA* strains were cultured o/n in LB broth, and RNA was extracted and used for cDNA synthesis and consecutive qPCR experiments. 16S rRNA expression levels were used for normalization. Depicted are the expression levels of *glgA* and *glgC* normalized to WT (=1). Shown is one representative assay of three independent biological replicates, consisting each of three technical replicates. Statistical analyses were performed by Student’s *t* test, and significances are indicated as follows: *, *P* < 0.05; **, *P* < 0.01; ***, *P* < 0.001.

The amount of stored glycogen is dependent on the abundance of synthesis enzymes (19), and glycogen synthesis in *S*. Typhimurium is mainly mediated by enzymes GlgA and GlgC (20). In E. coli, the main regulators for *glgA* and *glgC* transcription are the alarmones ppGpp and pppGpp [here referred to as (p)ppGpp] (21), which are induced during nutrient starvation by stringent response mediators RelA and SpoT. To elucidate whether *S*. Typhimurium Δ*fumABC* has an enhanced stringent response compared to *S*. Typhimurium WT, we made use of a dual-color reporter plasmid for relative quantification of *wraB* (=*wrbA* in E. coli) expression, which was recently used to determine the (p)ppGpp levels in E. coli (22). WrbA is known as a stationary-phase protein, whose expression is dependent on (p)ppGpp levels (23). Whereas initial studies identified WrbA as tryptophan-repressor-binding protein (24), other groups characterized it as a flavodoxin-like protein (25). We introduced the P*_wraB_*::sfGFP (superfolder green fluorescent protein) reporter plasmid into *S*. Typhimurium WT, Δ*fumABC*, and Δ*fumABC* Δ*glgA* strains and as negative control into *S*. Typhimurium Δ*relA* Δ*spoT*, a mutant strain deficient in (p)ppGpp synthesis (26), and analyzed the expression by flow cytometry (Fig. 5). To test reporter performance, stationary LB broth cultures of *S*. Typhimurium WT were subcultured in defined PCN (phosphate, carbon, nitrogen) minimal medium (MOPS-buffered minimal medium without limitation of phosphate, carbon, and nitrogen) with or without supplementation by Casamino Acids (Fig. 5A). Indeed, WT grown without an additional source of amino acids showed a higher sfGFP signal intensity than *S*. Typhimurium WT grown with amino acid supplementation, indicating higher (p)ppGpp levels.

Next, we determined sfGFP signal intensities of *S*. Typhimurium WT, Δ*fumABC*, and Δ*fumABC* Δ*glgA* strains harboring the respective reporter plasmid cultured in LB broth for 18.5 h as described before. Quantification of sfGFP intensity revealed higher values for *S*. Typhimurium Δ*fumABC* and Δ*fumABC* Δ*glgA* strains than for *S*. Typhimurium WT, whereas the negative-control *S*. Typhimurium Δ*relA* Δ*spoT* exhibited the lowest signal intensities (Fig. 5B and C). Additionally, transcript levels of *glgA* and *glgC* were determined (Fig. 5D). Strongly enhanced expression of *glgA* and *glgC* was detected for the Δ*fumABC* mutant compared to WT. For *S*. Typhimurium Δ*fumABC* Δ*glgA*, we detected only background signals for *glgA* but still highly increased expression levels of *glgC* compared to WT. In addition, glycogen accumulation in *S*. Typhimurium Δ*fumABC* was eliminated by further deletion of *relA* and *spoT* (Fig. S3). Thus, we propose that Δ*fumABC* enforces glycogen synthesis as a consequence of an early and strong stringent response, leading to high (p)ppGpp levels, which in turn raise the transcript and protein levels of GlgA and GlgC.

10.1128/mSphere.00796-19.3 FIG S3 Glycogen accumulation in Δ*fumABC* depends on (p)ppGpp-synthesizing enzymes. *S*. Typhimurium WT, Δ*fumABC*, Δ*relA spoT*, and Δ*fumABC* Δ*relA* Δ*spoT* strains were grown on LB agar plates for 18.5 h at 37°C. Potassium iodine staining was performed, and brownish color indicates intercalations of iodine with glycogen. Download FIG S3, TIF file, 0.7 MB. Copyright © 2019 Noster et al. 2019 Noster et al. This content is distributed under the terms of the Creative Commons Attribution 4.0 International license.

### Altered amounts of chemotaxis proteins in fumarase-deficient *S*. Typhimurium lead to increased counterclockwise (CCW) flagellar rotation.

**FIG 6:**
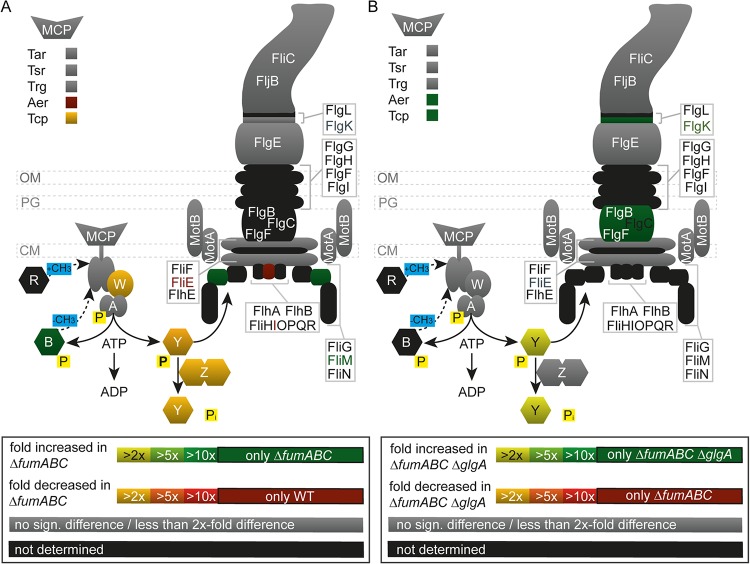
Deletion of fumarases affects amounts of flagellar and chemotaxis proteins, which are partly restored by deletion of *glgA*. *S*. Typhimurium WT, Δ*fumABC*, and Δ*fumABC ΔglgA* strains were grown aerobically in LB broth for 18.5 h at 37°C. For proteomic analyses, cells were harvested and lysed, and proteins were precipitated by 10% TCA. After trypsin digestion, peptides were analyzed by quantitative LC-MS^E^. Relative changes in protein abundance detected for Δ*fumABC* mutant compared to WT (A) and Δ*fumABC ΔglgA* mutant compared to Δ*fumABC* mutant (B) are indicated by color heat maps. Symbols in the upper left corner indicate abundance of methyl-accepting chemotaxis proteins (MCP). Symbols in the lower left corner represent chemotaxis proteins; letters indicate subunits (e.g., Y for CheY). P indicates phosphorylation. Data represent means from at least three biological replicates. Statistical analyses were performed by Student’s *t* test, and all data shown illustrate statistically significant differences between the two strains (*P* < 0.05).

Accumulation of (p)ppGpp can negatively affect motility, as recently described for E. coli (27). To explore this potential link, we analyzed proteomic data for modulation of chemotaxis and motility-related proteins (Fig. 6A). Decreased amounts of methyl-accepting chemotaxis proteins (MCP) and decreased abundance of CheY, CheZ, and CheW (2.14- to 3.86-fold) were detected in *S*. Typhimurium Δ*fumABC* compared to WT. In addition, CheB was found only in *S*. Typhimurium Δ*fumABC*. For *S*. Typhimurium Δ*fumABC* Δ*glgA*, a restoration of chemotaxis protein levels was detected for CheY. However, CheY abundance was still lower than in *S*. Typhimurium WT (Fig. 6B).

**FIG 7:**
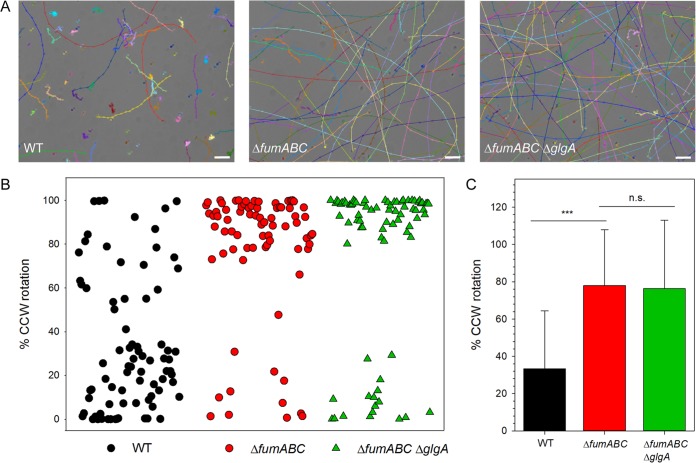
Fumarase deletions increase CCW flagellar rotation and running motility in a *glgA*-independent manner. (A) *S*. Typhimurium WT and mutant strains were cultured in LB broth for 18.5 h at 37°C and diluted 1:20 in PBS. Aliquots of the cultures were spotted onto microscope slides, and bacterial motion was recorded at 14 frames/s for 100 frames. For manual path tracking, the ImageJ plugin MTrackJ was used. Paths of individual *S*. Typhimurium WT, Δ*fumABC*, or Δ*fumABC* Δ*glgA* cells are indicated by various colors. Bars, 10 μm. (B) Bacteria were cultured for 18.5 h in LB, diluted 1:100 in PBS, subjected to shear force to remove flagellar filaments, and bound to polystyrene-coated coverslips. Rotating cells were selected, and rotation direction was recorded using standard brightfield microscopy for periods of 18 s. Rotation analyses were performed using the Matlab tool SpinningBug Tracker. By detection of the angle between the rotating bacteria and a reference axis, the rotation direction was calculated. Each symbol represents the analysis of one bacterial cell. The experimental setup and definitions are illustrated in Fig. S5 in the supplemental material. (C) Quantification of CCW bias of single *S*. Typhimurium cells. Means and standard deviations are shown for at least 75 cells per strain. Statistical analyses were performed by rank sum test, and significances are indicated as follows: ***, *P* < 0.001; n.s., not significant.

The amount of CheY influences the number of switching events of flagellar rotation direction (28). Thus, *S*. Typhimurium Δ*fumABC* might show an altered swimming behavior, and we analyzed swim patterns of bacteria grown overnight (o/n) in rich medium (Fig. 7A). Counterclockwise (CCW) flagellar rotation bundles flagella and results in straight swimming, while clockwise (CW) rotation leads to tumbling (29). *S*. Typhimurium WT showed short swimming paths alternating with tumbling, whereas *S*. Typhimurium Δ*fumABC* exhibited highly prolonged swimming paths and reduced tumbling events. Furthermore, the number of motile bacteria was higher than for WT. The motility patterns of Δ*fumABC* and Δ*fumABC* Δ*glgA* strains were similar.

To further analyze flagellar switching from CCW to CW rotation, we performed flagellar rotation analyses of *S*. Typhimurium WT, Δ*fumABC*, and Δ*fumABC* Δ*glgA* strains grown in rich medium by microscopic inspection of single bacterial cells fixed by one flagellum to a polystyrene-coated coverslip (30) (Fig. 7B and C). We observed a statistically significant increase of CCW flagellar rotation for *S*. Typhimurium Δ*fumABC*. Whereas *S*. Typhimurium WT had an average proportion of CCW rotation of 33%, the Δ*fumABC* strain spent 78% of time in CCW flagellar rotation. Although *S*. Typhimurium Δ*fumABC* Δ*glgA* exhibited partly normalized amounts of the chemotaxis protein CheY, there was still an increased proportion of CCW flagellar rotation comparable to that of *S*. Typhimurium Δ*fumABC*. Furthermore, the swimming behavior was not altered by *glgA* deletion, indicating that the amount of CheY necessary for normalization of switching events was not achieved in *S*. Typhimurium Δ*fumABC* Δ*glgA*.

Thus, we conclude that fumarase deletion in *S*. Typhimurium leads to a downregulation of chemotaxis proteins and by this to enhanced CCW flagellar rotation.

### The increased phagocytic uptake of fumarase-deficient *S*. Typhimurium is due to enhanced CCW flagellar rotation and partially depends on glycogen synthesis.

**FIG 8:**
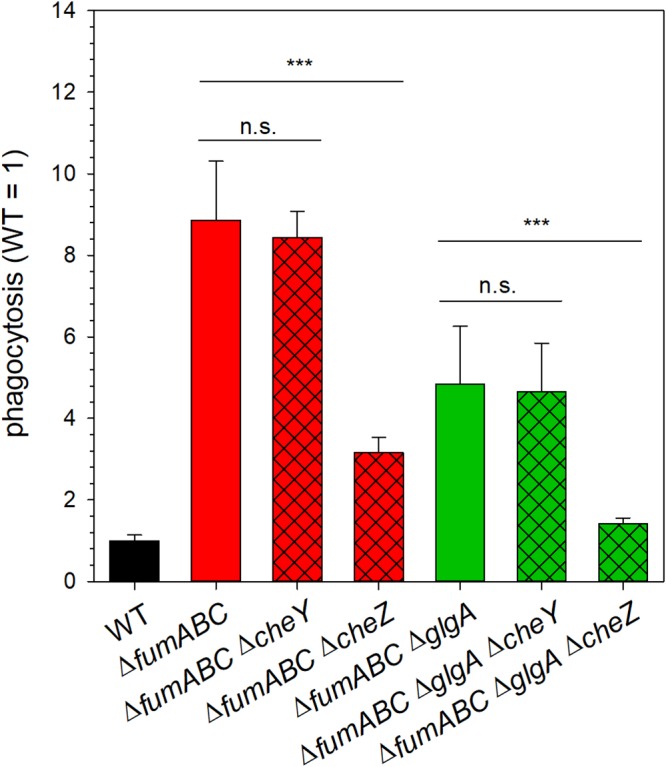
Increased phagocytic uptake of fumarase deletion strains is dependent on CCW flagellar rotation and glycogen synthesis. *S*. Typhimurium WT and various mutant strains as indicated were grown for 18.5 h in LB broth and used to infect RAW 264.7 macrophages at an MOI of 1. Infection was synchronized by centrifugation for 5 min, followed by incubation for 25 min at 37°C. Next, noninternalized bacteria were removed by washing and treatment with gentamicin at 100 μg/ml for 1 h and 10 μg/ml for the remaining time. Cells were lysed 2 h after infection by addition of 0.1% Triton X-100, and lysates were plated onto Mueller-Hinton agar plates to determine the CFU of internalized bacteria. Phagocytosis rates were determined as percentage of internalized bacteria relative to the used inoculum. Values were normalized to WT (=1), and means and standard deviations from three technical replicates are shown. Statistical analyses were performed by Student’s *t* test, and significances are indicated as follows: ***, *P* < 0.001; n.s., not significant.

Since bacterial motility can increase uptake of pathogenic bacteria by host cells (31–34), we hypothesized that the observed enhanced uptake of *fumABC* mutant strains by RAW 264.7 macrophages (12) could be caused by increased CCW flagellar rotation. To test this hypothesis, we introduced additional deletions of chemotaxis gene *cheY* or *cheZ* in the mutant strain. Whereas *cheY* deletion strains are locked in CCW flagellar rotation, Δ*cheZ* mutant strains are mainly locked in the CW state (35). The combination of Δ*cheY* and Δ*fumABC* did not alter phagocytic uptake, while the combination of Δ*cheZ* and Δ*fumABC* showed uptake only 3.15-fold higher than WT (Fig. 8).

Deletion of glycogen synthase partially normalized CheY levels but not the duration of CCW flagellar rotation in *S*. Typhimurium Δ*fumABC.* Thus, we expected an increased phagocytic uptake of the Δ*fumABC* Δ*glgA* double mutant as well. However, phagocytosis of *S*. Typhimurium Δ*fumABC* Δ*glgA* was 6.9-fold increased over WT, but significantly lower than uptake of *S*. Typhimurium Δ*fumABC* (Fig. 8). Complementation by plasmid-borne *glgA* again increased levels of phagocytosis (Fig. S4B). The *cheY* deletion did not change phagocytic uptake of *S*. Typhimurium Δ*fumABC* Δg*lgA*, while phagocytosis of *S*. Typhimurium Δ*fumABC* Δ*glgA* Δ*cheZ* was reduced (Fig. 8). These results demonstrate that high phagocytosis of fumarase deletion strains is due to CCW bias of flagellar rotation and is partially dependent on glycogen synthesis.

10.1128/mSphere.00796-19.4 FIG S4 Deletion of fumarases increases the phagocytic uptake by RAW 264.7 macrophages and is dependent on glycogen synthesis. RAW 264.7 macrophages were infected as described for Fig. 8. Values were normalized to WT (=1), and means and standard deviations from three technical replicates are shown. (A) RAW 264.7 macrophages were infected with WT, Δ*fumABC*, and Δ*fumABC* strains harboring plasmids carrying the intact genes *fumAC* or the *fumB* gene under the control of their native promoter or the empty vector, respectively. (B) RAW 264.7 macrophages were infected with WT, Δ*fumABC*, Δ*fumABC* Δ*glgA*, and Δ*fumABC* Δ*glgA* strains harboring a plasmid encoding wild-type GlgA under the control of its native promoter or the empty vector, respectively. The data are representative for three independent biological replicates. Statistical analyses were performed by Student’s *t* test, and significances are indicated as follows: **, *P* < 0.01; ***, *P* < 0.001; n.s., not significant. Download FIG S4, TIF file, 5.5 MB. Copyright © 2019 Noster et al. 2019 Noster et al. This content is distributed under the terms of the Creative Commons Attribution 4.0 International license.

**FIG 9:**
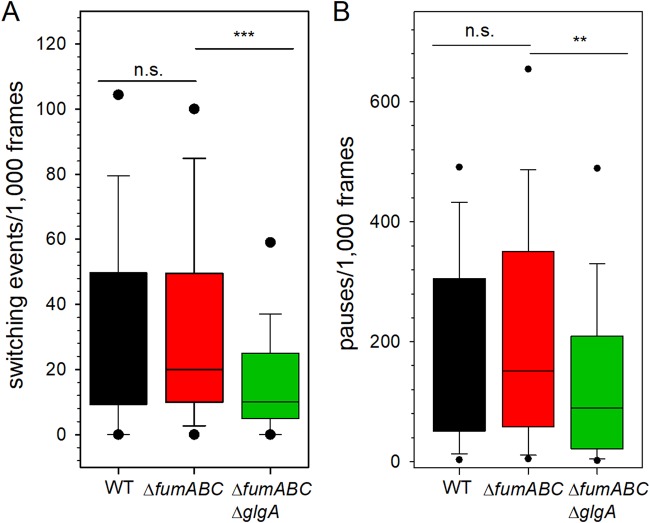
Deletion of fumarases and glycogen synthase affects the number of flagellar switching events and pauses. (A) Distribution of the number of switching events within 1,000 frames (total of 17.54 s) was calculated using flagellar rotation analysis (Fig. 7). (B) Distribution of pauses within 1,000 frames. Pause is defined as movement of the bacterial body less than 5°/frame. Values within the 5th/95th percentiles are excluded. Calculations are based on at least 75 analyzed bacteria. Statistical analyses were performed by rank sum test, and significances are indicated as follows: **, *P* < 0.01; ***, *P* < 0.001; n.s., not significant.

In order to elucidate which factors reduce the phagocytic uptake of *S*. Typhimurium Δ*fumABC* Δ*glgA* compared to Δ*fumABC*, we analyzed further characteristics of swimming behavior of both mutant strains. The frequency of switching events within 1,000 frames (17.71 s) was determined, and a switching event occurred if the flagellar rotation direction changed from CW to CCW or vice versa (Fig. S5). Compared to WT (median = 31 events), the switching rate was reduced in *S*. Typhimurium Δ*fumABC* (median = 20 events) but not in a significant manner (Fig. 9A). Even stronger reduction of switching events was determined for *S*. Typhimurium Δ*fumABC* Δ*glgA* (median = 10 events). Additionally the number of pauses, defined as rotation of the bacterial body of less than 5°/frame, was analyzed (Fig. 9B). Comparable to the number of switching events, WT had the highest number of pauses (median = 170.5), followed by *S*. Typhimurium Δ*fumABC* (151.5), but again there is no statistically significant difference between these two strains. A stronger reduction was observed for *S*. Typhimurium Δ*fumABC* Δ*glgA*; here, the number of pauses within 1,000 frames was reduced to 89.

10.1128/mSphere.00796-19.5 FIG S5 Method of flagellar rotation analysis. Bacteria were cultured for 18.5 h in LB and diluted 1:100 in PBS. (A) Bacteria were subjected to shearing to remove and shorten flagellar filaments. A small volume was given on an object slide and covered with a polystyrene-coated coverslip. Distance between object slide and coverslip was achieved by drops of vacuum grease. Dehydration or undertow was avoided by sealing the coverslip with “valap” (1:1:1 mixture of Vaseline, lanolin, and paraffin). Bacteria fixed with only one flagellum to the coverslip showed rotation of the body, which was recorded using the Axio Observer microscope with an AxioCam CCD camera (Zeiss) for periods of 17.54 s (frame rate: 57 frames/s). (B) Videos of rotating bacteria were processed using ImageJ, and rotation analyses were performed using the tool SpinningBug Tracker. By detection of the angle between the rotating bacteria and a reference axis, the rotation direction was calculated. (C) If there was a change of degree of more than 5°, it was defined as rotation. The visible clockwise rotation of the bacterial body results from counterclockwise rotation of a flagellum and vice versa. Download FIG S5, TIF file, 0.4 MB. Copyright © 2019 Noster et al. 2019 Noster et al. This content is distributed under the terms of the Creative Commons Attribution 4.0 International license.

In conclusion, *S*. Typhimurium Δ*fumABC* showed strongly increased CCW bias and fewer switching events than *S*. Typhimurium WT. These factors influence the interaction with host cells, such as increasing phagocytic uptake by macrophages. Further deletion of *glgA* in the Δ*fumABC* strain did not reduce time spent in CCW flagellar rotation but decreased the number of switching events, resulting in reduced phagocytic uptake.

## DISCUSSION

Our work investigated the effect of perturbation of the TCA cycle of *S*. Typhimurium on basic cellular functions and pathometabolism. Enzymes harboring iron-sulfur clusters, i.e., fumarases and aconitases, are of particular sensitivity toward ROS attacks, which are a consequence of antibiotic treatment or immune responses in phagocytic host cells. We were interested in physiological changes and aberrant virulence properties upon ROS-dependent inactivation of metabolic enzymes and focused on a mutant strain defective in all fumarase isoforms. By deploying proteomics and metabolomics, we determined that defects in fumarases biased carbon fluxes toward enhanced glycogen synthesis, likely due to elevated (p)ppGpp levels in the mutant strain. Furthermore, proteomics revealed reduced abundances of chemotaxis proteins in *S*. Typhimurium Δ*fumABC*. Analysis of flagellar rotation and swim patterns showed increased CCW bias, raising the contact frequency of *S*. Typhimurium and host cells, thus leading to enhanced phagocytic uptake by macrophages. Deletion of glycogen synthase GlgA relieved the metabolic perturbations but not the aberrant motility phenotype. However, phagocytic uptake was decreased.

Our metabolomics data demonstrated higher accumulation of G6P, F6P, and S7P for *S*. Typhimurium Δ*fumABC* than for WT, and deletion of glycogen synthase again normalized the metabolic flux through glycolysis and PPP (Fig. 4). Thus, the increased concentrations of these metabolites were caused by enhanced glycogen synthesis in *S*. Typhimurium Δ*fumABC* due to changes in carbon fluxes. Accumulation of the respective metabolites was also observed for E. coli with truncated CsrA, the main component of the carbon storage system (36, 37). As *csrA* deletion strains accumulate large amounts of glycogen as well, our results indicate that the observations obtained for E. coli Δ*csrA* are also consequences of the massive remodeling of the carbon metabolism due to enhanced glycogen synthesis. However, a role of CsrA was reported not only in the context of posttranscriptional regulation of carbon metabolism, and in particular glycogen metabolism, but also for chemotaxis proteins, flagellar subunits, and proteins involved in virulence functions (38, 39). Thus, the involvement of CsrA as an inducer of phenotypes of *S*. Typhimurium Δ*fumABC* is conceivable. While glycogen accumulation indicates very low levels of CsrA, mutant strains with truncated CsrA showed increased levels of Pgm and reduced levels of especially PfkA in E. coli (36), observations which are contradictory to our results. However, most studies on *csrA* mutant strains were performed with bacteria grown in minimal medium or at early growth phases (36). Thus, we cannot exclude a role of CsrA in the enhancement of glycogen synthesis for *S*. Typhimurium Δ*fumABC*, yet we do not expect CsrA to be the sole regulating factor.

In contrast, (p)ppGpp was shown to be the most important factor influencing glycogen synthesis, at least in E. coli (40). (p)ppGpp is known to enhance the expression of *glgA* and *glgC* but not *glgB* during stringent response (19). Indeed, we detected GlgA and GlgC only in *S*. Typhimurium Δ*fumABC* (Fig. 2D). Using a dual-color reporter system with P*_wraB_* controlling sfGFP expression, we detected increased fluorescence intensities for *S*. Typhimurium Δ*fumABC* and Δ*fumABC* Δ*glgA* compared to WT. The promoter of *wrbA* was used in several studies for the indirect quantification of (p)ppGpp (22, 41). Furthermore, by proteomic analyses we detected increased abundances of WrbA in the Δ*fumABC* strain (3.78-fold; see Table S2 in the supplemental material), supporting our results obtained by flow cytometry. Taken together, we hypothesize that a fumarase deletion strain increases *glgA* and *glgC* expression in a (p)ppGpp-dependent manner.

The main inducing factors for (p)ppGpp synthesis by RelA and SpoT are amino acid and carbon source limitations (23). Using LB broth, amino acid limitations are unlikely at early growth phase. Several studies showed that increase of (p)ppGpp levels can be induced by diauxic shifts, for example, from glucose to succinate (42). Considering the high accumulation of fumarate, the use of the TCA cycle intermediate as carbon source is conceivable. An indicator for this model is the slightly increased abundance of aspartase AspA in *S*. Typhimurium Δ*fumABC* (1.5-fold), catalyzing the reversible reaction from fumarate and ammonia to aspartate (43). Indeed, metabolomic data showed a 10-fold-larger amount of aspartate in the mutant strain, which serves as the substrate for a range of metabolic pathways (44). Furthermore, two studies indicated that high fumarate accumulation led to use of fumarate as an alternative electron acceptor, despite the presence of oxygen (15, 45). However, our proteomic data gave no hints for fumarate respiration (i.e., fumarate reductase FrdABCD) in the mutant strain but rather indicated utilization of fumarate as a carbon source. Fumarate metabolism possibly leads to a physiological situation similar to exponential- to stationary-phase transition and therefore increased (p)ppGpp levels, as discussed for E. coli (15, 22).

Absence of fumarases led to enhanced CCW flagellar rotation and a prolonged phase of running movement, resulting in increased uptake by RAW 264.7 macrophages (Fig. 8). The impact of CCW flagellar rotation during the infection process was discussed in several prior publications (31–33). In these studies, CCW flagellar rotation and the resulting smooth swimming phenotype were linked to enhanced frequencies of bacterial contact with host cells, prolonged duration of adhesion, and increased numbers of phagocytic uptake events. Further deletion of *glgA* in *S*. Typhimurium Δ*fumABC* partly restored CheY levels, and we observed reduced uptake of *S*. Typhimurium Δ*fumABC* Δ*glgA* by macrophages. As we determined a strongly decreased number of switching events for the *glgA*-deficient strain but high frequency of phases of CCW flagellar rotation, the logical consequence is that duration of phases of CW flagellar rotation after switching events is longer for *S*. Typhimurium Δ*fumABC* Δ*glgA* than for the Δ*fumABC* strain. This effect might be accompanied by the reduced number of pause events observed for *S*. Typhimurium Δ*fumABC* Δ*glgA* in comparison to Δ*fumABC* and WT strains and could lead to changes in frequency or duration of contacts between *S*. Typhimurium and host cells.

**FIG 10:**
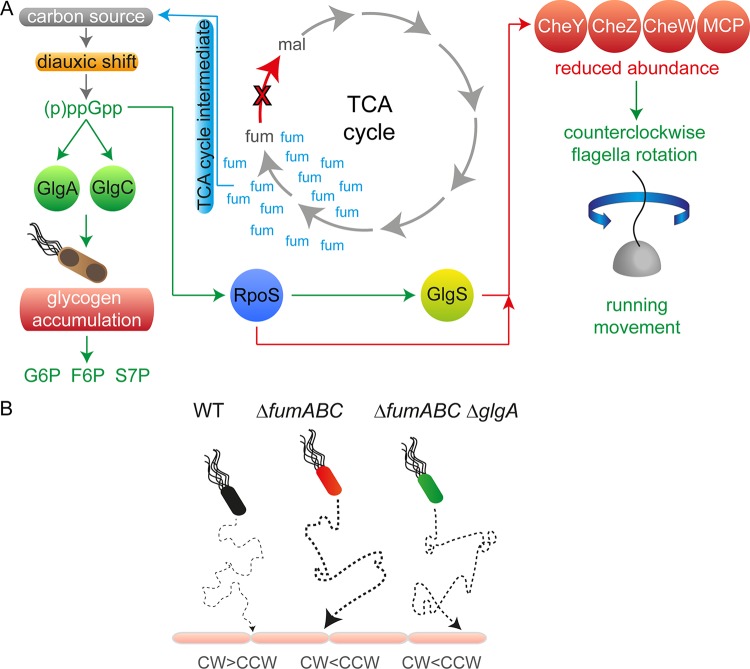
Model summarizing effects of fumarase deletion on *S*. Typhimurium pathophysiology. (A) Fumarase deletion leads to fumarate accumulation. If present in high concentrations, it is used as carbon source, which induces a kind of diauxic shift and increased (p)ppGpp concentrations. The alarmone accelerates the expression of glycogen-synthesizing enzymes, and glycogen accumulation occurs. This metabolic shift is accompanied by increased concentrations of glycolytic and PPP intermediates. (p)ppGpp strengthens RpoS biosynthesis, which in turn enhances GlgS abundance. GlgS and RpoS reduce expression of flagellar genes, leading to decreased CheY levels and enhanced CCW flagellar rotation. (B) Swimming behavior biases phagocytic uptake rates. WT flagella are rotating mainly CW, resulting in tumbling. Due to reduced numbers of cell contacts with the host cell, phagocytic uptake is low. *S*. Typhimurium Δ*fumABC* and Δ*fumABC* Δ*glgA* strains show both increased CCW rotation and running movement, raising the number of host cell contacts and thereby phagocytic uptake. *S*. Typhimurium Δ*fumABC* Δ*glgA* exhibits prolonged CW periods, which possibly leads to slight reduction of phagocytosis rates.

To conclude, our results demonstrate that accumulation of fumarate due to fumarase deletion leads to induction of glycogen synthesis by enhanced (p)ppGpp concentrations (Fig. 10). This might be triggered by utilization of fumarate as carbon source, causing an exponential- to stationary-phase transition-like physiological state during early stationary growth phase. Additionally, we revealed that the increased phagocytic uptake of the fumarase deletion strain is caused by enhanced CCW flagellar rotation, which is the consequence of reduced CheY abundance. Further deletion of *glgA* normalized metabolic fluxes and restored abundance of the chemotaxis protein in part but did not change CCW bias of flagellar rotation. However, *glgA* deletion led to reduced phagocytic uptake by RAW 264.7 macrophages, possibly due to prolonged periods of CW flagellar rotation. Our work demonstrates that perturbations of the carbon fluxes in the TCA cycle lead to dramatic changes in *S*. Typhimurium physiology and affect the interaction of this pathogen with host cells.

## MATERIALS AND METHODS

### Bacterial strains.

Salmonella enterica serovar Typhimurium NCTC 12023 was used as the wild-type strain (WT), and isogenic mutant strains were constructed by λ Red-mediated mutagenesis (Table 1) (46). Primers and plasmids required for mutagenesis, removal of resistance cassettes, and checking for the correct insertion are listed in Table 2 and in Table S4 in the supplemental material. Transfer of mutant alleles into a fresh strain background or for combination with other mutations occurred via P22 transduction. Both methods are described in the work of Popp et al. (47).

**TABLE 1:** Bacterial strains used in this study

Designation	Genotype	Relevant defect(s)	Source or reference
NCTC 12023	Wild type		NCTC, Colindale, United Kingdom
MvP1564	Δ*fumAC*::FRT^*a*^ Δ*fumB*::FRT	Fumarases A, B, and C	12
MvP2042	Δ*glgA*::*aph*	Glycogen synthase	This study
MvP2046	Δ*fumAC*::FRT Δ*fumB*::FRT Δ*glgA*::*aph*	Fumarases A, B, and C; glycogen synthase	This study
MvP1209	Δ*cheY*::*aph*	Chemotaxis protein CheY	This study
MvP1741	Δ*fumAC*::FRT Δ*fumB*::FRT Δ*cheY*::*aph*	Fumarases A, B, and C; chemotaxis protein Y	This study
MvP1527	Δ*cheZ*::*aph*	Protein phosphatase CheZ	This study
MvP1739	Δ*fumAC*::FRT Δ*fumB*::FRT Δ*cheZ*::*aph*	Fumarases A, B, and C; protein phosphatase CheZ	This study
MvP2691	Δ*fumAC*::FRT Δ*fumB*::FRT Δ*glgA*::FRT	Fumarases A, B, and C; glycogen synthase	This study
MvP2692	Δ*fumAC*::FRT Δ*fumB*::FRT Δ*glgA*::FRT Δ*cheY*::*aph*	Fumarases A, B, and C; glycogen synthase; chemotaxis protein CheY	This study
MvP2693	Δ*fumAC*::FRT Δ*fumB*::FRT Δ*glgA*::FRT Δ*cheZ*::*aph*	Fumarases A, B, and C; glycogen synthase; protein phosphatase CheZ	This study
MvP1517	Δ*gltA*::FRT	Citrate synthase	This study
MvP1576	Δ*acnA*::FRT Δ*acnB*::FRT	Aconitases A and B	12
MvP1482	Δ*icdA*::FRT	Isocitrate dehydrogenase	This study
MvP1165	Δ*sucAB*::FRT	α-Ketoglutarate dehydrogenase	This study
MvP1523	Δ*sdhCDAB*::FRT	Succinate-dehydrogenase subunits A, B, C, and D	12
MvP1524	Δ*sucCD*::FRT	Succinate-CoA ligase	This study
MvP1484	Δ*mdh*::FRT	Malate dehydrogenase	This study
MvP2862	Δ*relA*::FRT Δ*spoT*::*aph*	(p)ppGpp synthetases 1 and 2	This study
MvP2863	Δ*fumAC*::FRT Δ*fumB*::FRT Δ*relA*::FRT Δ*spoT*::*aph*	Fumarases A, B, and C; (p)ppGpp synthetases 1 and 2	This study
Donor strains used for P22 transduction			
MvP1209	Δ*cheY*::*aph*	Chemotaxis protein CheY	31
MvP1527	Δ*cheZ*::*aph*	Protein phosphatase CheZ	31
KT9616	Δ*relA*::*aph*	(p)ppGpp synthetase 1	Karsten Tedin
KT9684	Δ*relA*::FRT Δ*spoT*::*aph*	(p)ppGpp synthetases 1 and 2	Karsten Tedin
MvP2042	Δ*glgA*::*aph*	Glycogen synthase	This study

a FRT, FLP recognition target.

**TABLE 2:** Plasmids used in this study

Plasmid	Relevant characteristic(s)	Reference(s)
pKD46	Red-expressing vector, *ts*, Amp^r^	46
pWRG730	Red-expressing vector, *ts*, Cm^r^	54
pKD13	Template plasmid containing kanamycin cassette, recombinase target sites (FRT), Amp^r^ Kan^r^	46
pE-FLP	Flippase-expressing vector, *ts*, Amp^r^	55
pCP20	Flippase-expressing vector, *ts*, Amp^r^	46
pWSK29	Low-copy-number cloning vector, Amp^r^	56
pWSK30	Low-copy-number cloning vector, Amp^r^	56
p3752	pWSK30::*P_fumA_*::*fumAC*, Amp^r^	This study
p3756	pWSK30::*P_fumB_*::*fumB*, Amp^r^	This study
p4763	pWSK29::*P_glgB_*::*glgBXCAP*, Amp^r^	This study
p4889	P_EM7_::DsRed P*_uhpT_*::sfGFP	48; Röder and Hensel, submitted for publication
p5371	P_EM7_::DsRed P*_wraB_*::sfGFP	This study

10.1128/mSphere.00796-19.9 TABLE S4 Oligonucleotides used for mutagenesis. Download Table S4, DOCX file, 0.01 MB. Copyright © 2019 Noster et al. 2019 Noster et al. This content is distributed under the terms of the Creative Commons Attribution 4.0 International license.

### Construction of plasmids.

For generation of p3752 and p3756, wild-type promoters and coding sequences of *fumAC* and *fumB* were amplified with primers listed in Table S4. After digestion with NotI and XhoI or ApaI and XhoI, respectively, the gene products were ligated into the low-copy-number plasmid pWSK30 and transformed in E. coli DH5α. Positive clones were confirmed with primers listed in Table S4. The plasmids were isolated and transformed in the Δ*fumAC* or Δ*fumB* deletion strain.

For construction of p4763, the promoter and sequence of *glgBXCAP* as well as the vector pWSK29 were amplified by PCR using primers listed in Table S4. The obtained PCR fragments were assembled by Gibson assembly according to the manufacturer’s protocol (New England BioLabs [NEB]). Sequence-confirmed plasmids were transformed in the Δ*fumABC* Δ*glgA* deletion strain.

Generation of the reporter plasmid p5330 was performed as described previously (48). Briefly, plasmid p4889 (P_EM7_::DsRed P*_uhpT_*::sfGFP) was used as vector. The *uhpT* promoter was replaced by the promoter fragment of *wraB* by Gibson assembly of fragments generated by PCR. Primers for fragment generation are listed in Table S4. Sequence-confirmed plasmids were transformed in *S*. Typhimurium WT, Δ*fumABC*, Δ*fumABC* Δ*glgA*, and Δ*relA* Δ*spoT* strains.

### GC-MS sample preparation and measurement.

Culture of strains and cell harvest occurred as described in the work of Noster et al. (12). In short, each strain was cultured for 18.5 h at 37°C in 25 ml LB broth with agitation at 180 rpm. For measurements of metabolites in bacterial cells, 5 ml of cultures was transferred onto Durapore polyvinylidene difluoride (PVDF) filter membranes (Merck, Darmstadt, Germany) with a pore size of 0.45 μm by suction. After washing with PBS, cells were scraped from the filter into 1 ml of fresh PBS, pelleted, and shock-frozen in liquid nitrogen. Afterward, samples were freeze-dried and their dry weights were determined. Metabolome analysis of the TCA cycle mutant strains was performed by GC-MS using protocols according to the work of Plassmeier et al. (49) and Noster et al. (12). In short, for metabolite extraction 1 ml 80% methanol containing 10 μM ribitol (RI; internal standard) was added to dried samples, and for cell disruption, 500 mg acid-washed glass beads (Sigma-Aldrich, USA) and a homogenizer (Precellys; Peqlab) were used. After centrifugation, supernatants were evaporated in a nitrogen stream. For derivatization, 50 μl of 20 mg/ml methoxylamine hydrochloride in pyridine and *N*-methyl-*N*-(trimethylsilyl)-trifluoroacetamide was added successively to each sample and incubated with constant stirring at 37°C for 90 min or 30 min, respectively. RI standard was added and incubated for a further 5 min. Samples were centrifuged, and supernatants were used for GC-MS measurement using a TraceGC gas chromatograph equipped with a PolarisQ ion trap and an AS1000 autosampler (Thermo Finnigan, Dreieich, Germany) according to the work of Plassmeier et al. (49). Metabolite quantities were normalized to ribitol and dry weights of used samples as described in the work of Plassmeier et al. (49). Mean relative pool size changes of the mutant strains compared to WT were calculated, and only those data with an error probability (Student’s *t* test) of less than 0.05 were used for further interpretation.

### Proteome profiling by nano LC-MS measurement.

Bacteria were cultured as described for the metabolite profiling. Sample preparation and liquid chromatography (LC)-MS measurement were performed according to the work of Noster et al. (12). In short, cells from 50 ml overnight (o/n) culture were pelleted, suspended, and washed twice with PBS. Pelleted bacteria were resuspended in lysis buffer (50 mM Tris, pH 8.5, 1% SDS, protease inhibitor). Cell disruption occurred with zirconia-silica beads and a cell homogenizer. Cell debris was removed by centrifugation, and proteins were precipitated with TCA. Protein pellets were washed with acetone, dried, and used for the following sample preparation, proteomic digestion, and nano LC-MS measurement as described in the work of Noster et al. (12).

### Gentamicin protection assay.

Culture and infection of RAW 264.7 macrophages were performed as described in the work of Popp et al. (47). Briefly, RAW 264.7 macrophages were infected with *S*. Typhimurium o/n cultures at an MOI of 1 and centrifuged for 5 min at 370 × *g*. The infection proceeded for a further 25 min. Cells were washed three times with PBS, and extracellular, nonphagocytosed bacteria were killed by incubation with medium containing gentamicin (100 μg/ml for hour 1, 10 μg/ml for hour 2). At 2 h postinfection (p.i.), cells were washed three times with PBS and lysed by addition of 0.1% Triton X-100 in PBS. Serial dilutions of the inoculum and lysates were plated on Mueller-Hinton II agar plates and incubated o/n at 37°C. Phagocytosis rates were determined as percentage of internalized bacteria relative to the inoculum.

### Qualitative and quantitative determination of glycogen content.

Qualitative determination of glycogen contents of bacterial cultures occurred as described in the work of Govons et al. (18). Bacterial cultures were streaked on LB agar plates and incubated o/n at 37°C. Ten milliliters of Lugol’s iodine solution (Roth) was added to the plate and incubated 1 min at room temperature (RT). The iodine solution was discarded, and the plates were photographed immediately.

Quantification of glycogen contents occurred according to the protocol of Fung et al. (50). Of each strain, cells of 5 ml o/n culture were pelleted by centrifugation (13,000 × *g*, 10 min, 4°C), resuspended in 50 mM Tris-acetate-EDTA (TAE) buffer, and pelleted again. Cells were resuspended in 1.25 ml sodium acetate buffer (200 mM, pH 4.6), and the suspension was added to 500 mg glass beads and disrupted by three cycles, each of 1 min with maximal speed, using a Vortex Genie 2, equipped with an attachment for microtubes (Scientific Industries). After centrifugation, supernatants were incubated for 20 min at 80°C for denaturation of endogenous enzymes. For each strain, 60 μl lysate was incubated with 6 μl amyloglucosidase (200 U/ml; Sigma-Aldrich) (quantification of glucose stored as glycogen and free glucose) or 6 μl water (quantification of free glucose), respectively. After incubation for 30 min at 50°C, 50 μl of each sample was transferred into a 96-well plate in technical duplicates. Two hundred fifty microliters HK reagent (Sigma-Aldrich) was added to each sample, and OD_340_ was determined in 10-min intervals for 1 h. A standard curve with different dilutions of a glucose solution was used for extrapolation of the determined data. For relative quantification of the glycogen amount, maximal values obtained for free glucose were subtracted from maximal values obtained from free glucose and glycogen and normalized to the OD_600_ of the bacterial culture.

### TEM analysis.

For TEM analyses of bacteria, *S*. Typhimurium was grown o/n at 37°C in LB broth with aeration. Cells were harvested for 2 min at 1,250 × *g*. The pellet was resuspended in buffer (0.2 M HEPES, pH 7.4, 5 mM CaCl_2_), and bacteria were fixed by addition of glutaraldehyde (Electron Microscopy Sciences) in buffer to a final concentration of 2.5% for 1 h at 37°C. After fixation, bacteria were washed several times in buffer and harvested for 5 min at 625 × *g*. The pellet was gently resuspended in liquid 2% low-melting-point (LMP) agarose prewarmed to 37°C in buffer and incubated for 10 min at 37°C. Bacteria in agarose were repelleted for 1 min at 1,250 × *g* and cooled down to 4°C until agarose was solid. The agarose block containing the bacteria was cut into small cubes (maximum, 1 mm^3^), and cubes were postfixed with 2% osmium tetroxide (Electron Microscopy Sciences) in buffer containing 1.5% potassium ferricyanide (Sigma) and 0.1% ruthenium red (AppliChem) for 1.5 h at 4°C in the dark. After several washing steps, bacteria were dehydrated in a cold graded ethanol series and finally rinsed in anhydrous ethanol at RT twice. The agarose cubes were flat-embedded in Epon 812 (Serva). Serial 70-nm sections were generated with an ultramicrotome (Leica EM UC6) and collected on Formvar-coated EM copper grids. After staining with uranyl acetate and lead citrate, bacteria were observed with a TEM (Zeiss EFTEM 902 A), operated at 80 keV and equipped with a 2K wide-angle slow-scan charge-coupled device (CCD) camera (TRS, Moorenweis, Germany). Images were taken with the software ImageSP (TRS Image SysProg, Moorenweis, Germany).

### Flagellar rotation analysis.

Flagellar rotation was determined as illustrated in Fig. S5. Bacteria were cultured for 18.5 h in LB, diluted 1:100 in PBS, and subjected to shear force by pressing the suspension eight times through a syringe equipped with a 24-gauge cannula. Fifteen microliters of sample was placed onto a microscope slide and covered with a coverslip spin-coated with polystyrene, on which three small drops of vacuum grease were spotted to achieve an optimal distance allowing free movement of *S*. Typhimurium. Sealing the cover slip with a 1:1:1 mixture of Vaseline, lanolin, and paraffin avoided suction. Rotating cells, bound with their flagellar filaments to the coverslip, were selected, and rotation direction was recorded using the Axio Observer microscope with an AxioCam CCD camera (Zeiss) for periods of 18 s (using a 100× alpha-Plan-Apochromat objective, 1.6× Optovar, 10 ms exposure, frame rate of 57/s). After image processing with Fiji (conversion to 8-bit format; background subtraction: rolling ball radius 20 pixels; cropping down to rotation disk; contrast enhancement: saturated pixels 0.0%, normalized; downsizing: width 10 pixels, constraint of aspect ratio, averaged, interpolated; Gaussian Blur: sigma 0.5; contrast enhancement), rotation analyses were performed using the tool SpinningBug Tracker (user-written software available from the authors upon request, Matlab 7.17 [R2012a]). By detection of the angle between the rotating bacteria and a reference axis, the rotation direction was calculated. CCW rotation of the bacterial body has to be interpreted as CW rotation of the flagellum and vice versa. Rotations of less than 5°/frame were defined as pause. Bacteria rotating with speeds of >180°/frame were excluded, due to limited time-resolution. Switching events were defined as changes from CW to CCW rotation and vice versa.

### Swimming path analysis.

Bacteria were cultured for 18.5 h with aeration in LB and diluted 1:20 in PBS. The assembly of microcopy slide, sample, and coverslip was similar to that described for the flagellar rotation analysis, but without prior coating of the coverslip with polystyrene. The swimming bacteria were recorded for 100 frames (14 frames/s) using a 40× LD Plan-Neofluar objective, 1.6 Optovar, 10 ms exposure. Visualization of swimming paths was performed with ImageJ, using the plug-in MTrackJ with time step size 5 frames, snap range 25 × 25 pixels (51).

### qPCR.

For RNA preparation by the hot-phenol method, bacteria were cultured for 18.5 h in LB with aeration. Bacteria at a number of 1.2 × 10^9^ were pelleted, treated with stop solution (95% ethyl alcohol [EtOH], 5% phenol saturated with 0.1 M citrate buffer, pH 4.3) (Sigma-Aldrich), and snap-frozen in liquid nitrogen. All subsequent steps were conducted as described in detail in the work of Noster et al. (48) according to protocols from Mattatall and Sanderson (52) and Sittka et al. (53). For cDNA synthesis, the RevertAid first-strand cDNA synthesis kit (ThermoFisher) was used, applying 1 μg RNA and random hexamer primers. qPCR was performed using the Maxima SYBR green/fluorescein qPCR master mix (ThermoFisher) and an iCycler equipped with the MyiQ module (Bio-Rad). Data were normalized to expression levels of a housekeeping gene (16S rRNA) and calculated relative to primer efficiencies, which were determined before using serial dilutions of cDNA. Used oligonucleotides are listed in Table S4.

### Flow cytometry analyses.

*S*. Typhimurium strains harboring p5330 were grown in LB broth at 37°C with aeration for 18.5 h, diluted 1:1,000 in FACS buffer (1% BSA in PBS, 1 mM EDTA, 20 mM HEPES, pH 7.2, 50 mM NH_4_Cl), and subjected to flow cytometry on an Attune NxT instrument (Thermo Fisher Scientific). The intensity of the sfGFP fluorescence per gated *S*. Typhimurium cell of 10,000 bacteria with constitutive red fluorescence was recorded, and *x* medians for sfGFP intensities were calculated.
