# Supplementary figures and images for: Blocks in Tricarboxylic Acid Cycle of Salmonella enterica Cause Global Perturbation of Carbon Storage, Motility, and Host-Pathogen Interaction
Source: mSphere. 2019 Dec 11;4(6):e00796-19. doi: 10.1128/mSphere.00796-19 (PMC6908425; doi:10.1128/mSphere.00796-19)

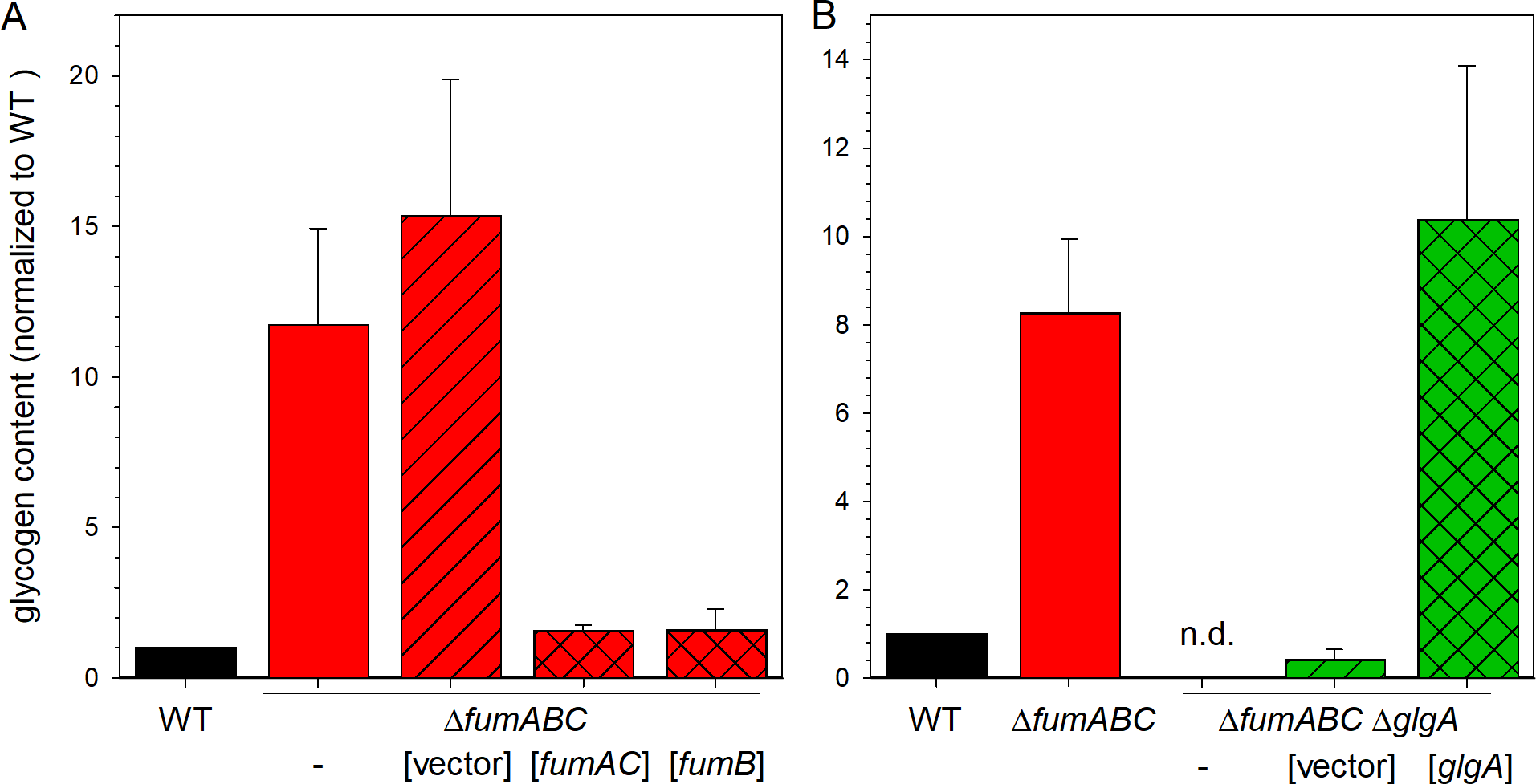

Supplement: FIG S1 [file mSphere.00796-19-sf001.tif]

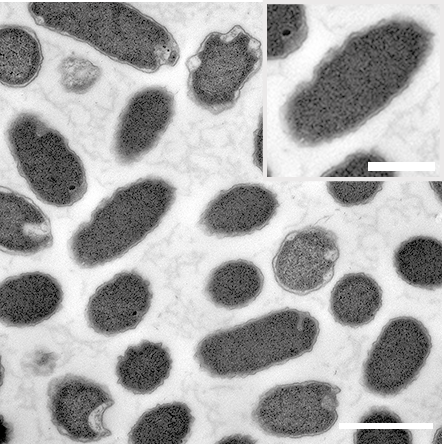

Supplement: FIG S2 [file mSphere.00796-19-sf002.tif]

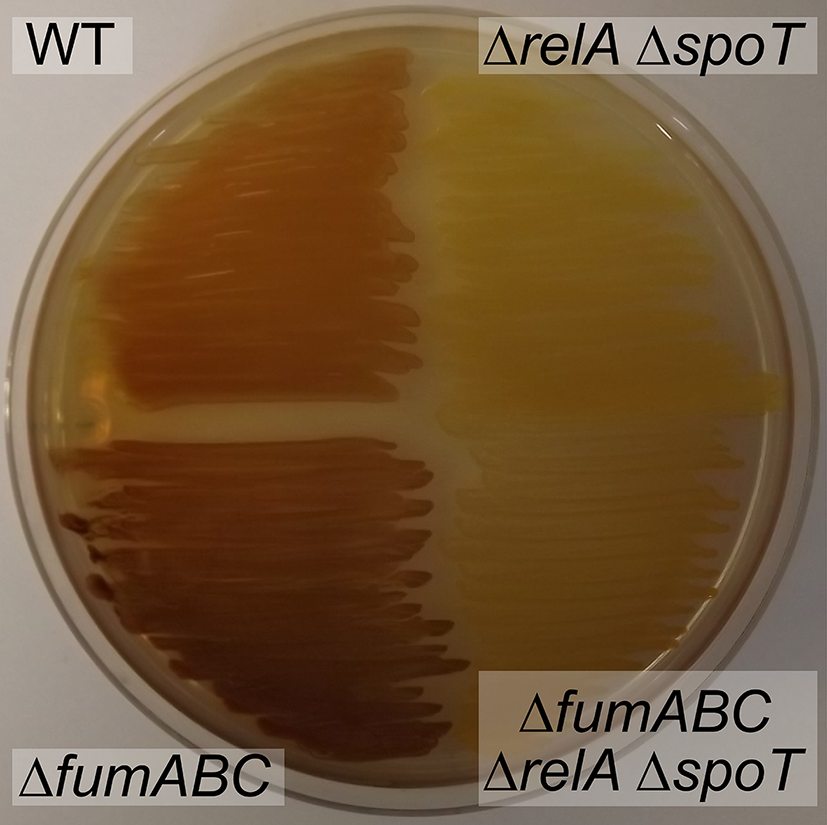

Supplement: FIG S3 [file mSphere.00796-19-sf003.tif]

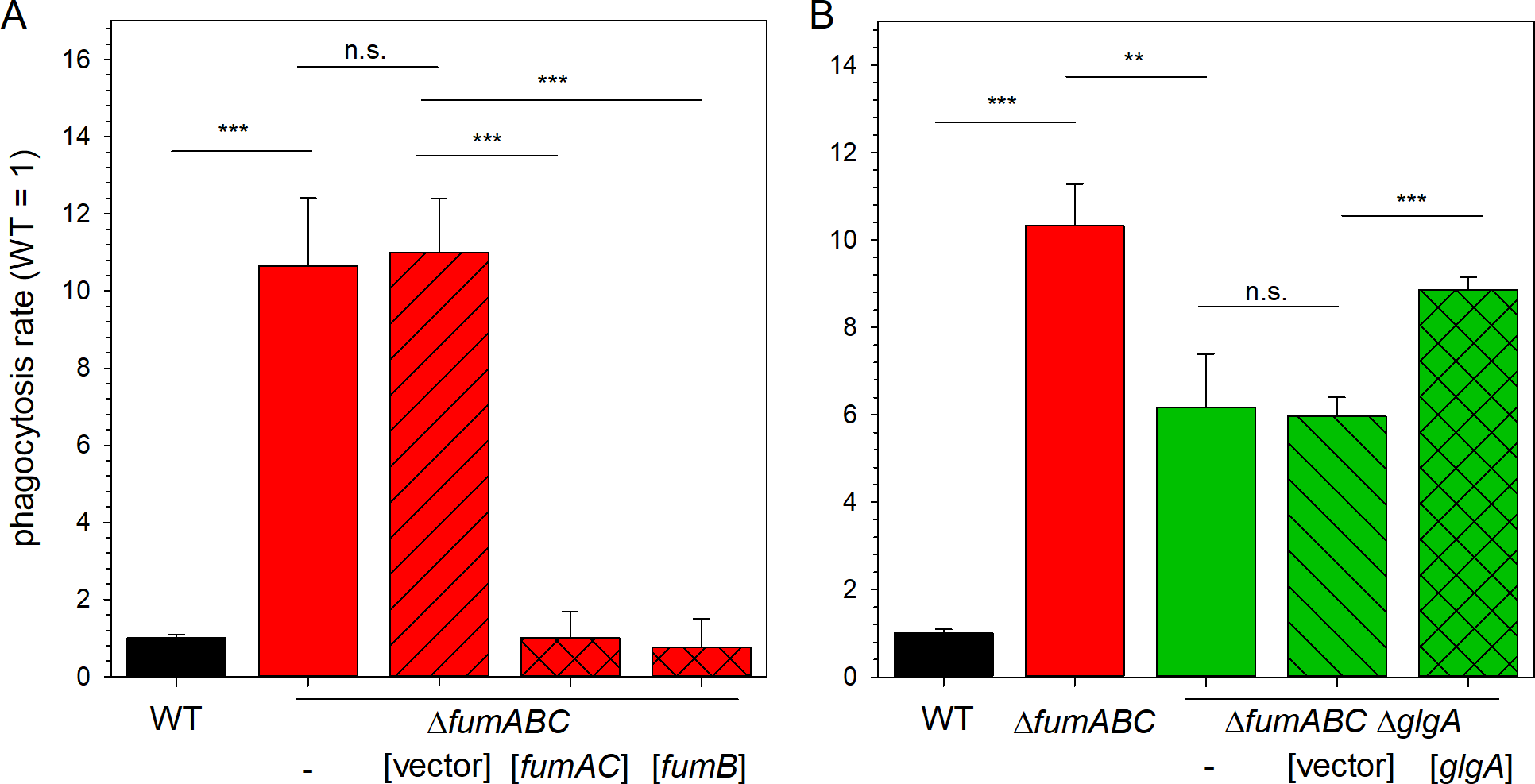

Supplement: FIG S4 [file mSphere.00796-19-sf004.tif]

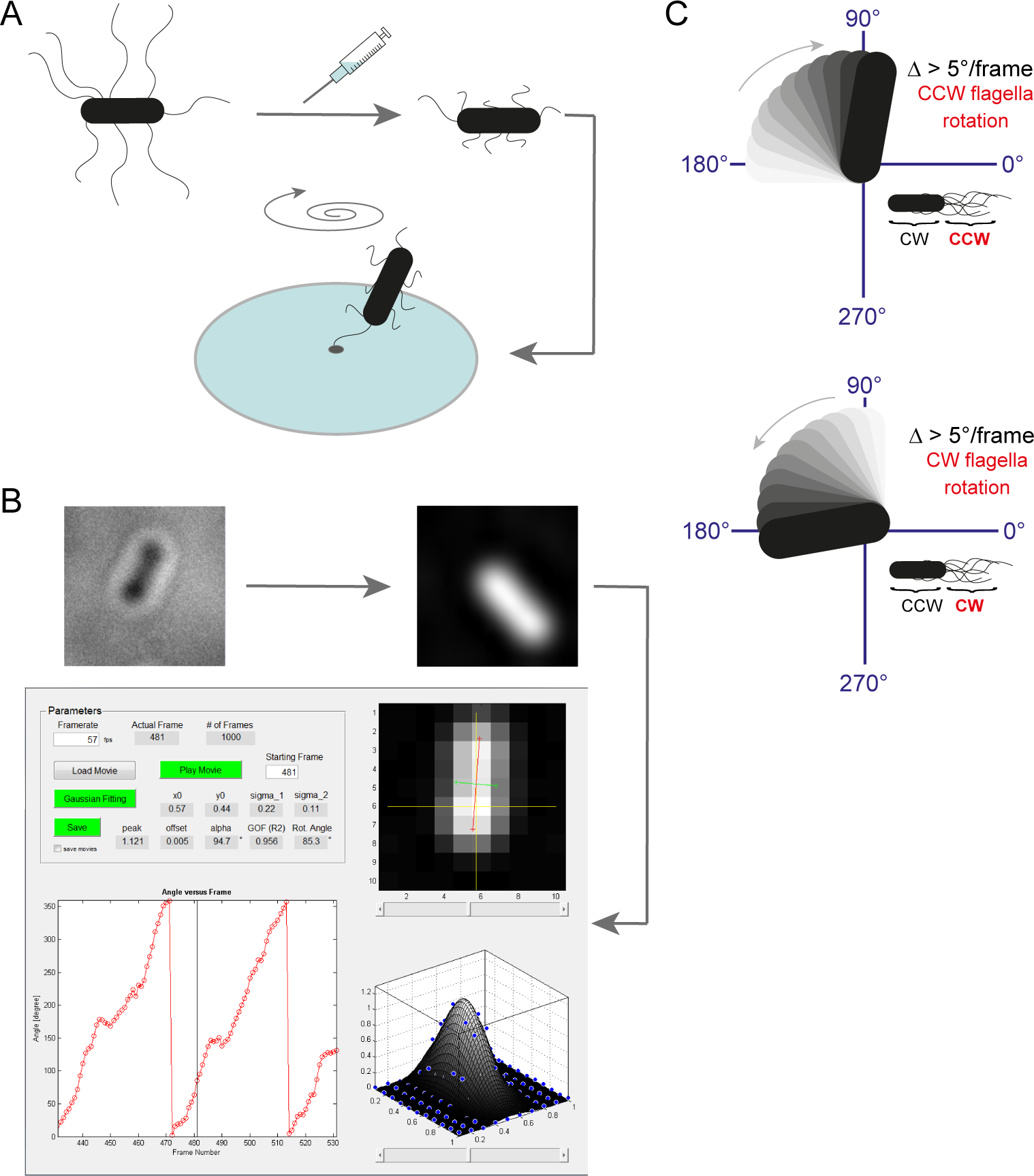

Supplement: FIG S5 [file mSphere.00796-19-sf005.tif]
