# Supplementary material for: Blocks in Tricarboxylic Acid Cycle of Salmonella enterica Cause Global Perturbation of Carbon Storage, Motility, and Host-Pathogen Interaction
Source: mSphere. 2019 Dec 11;4(6):e00796-19. doi: 10.1128/mSphere.00796-19 (PMC6908425; doi:10.1128/mSphere.00796-19)
Supplement: TABLE S4 [file mSphere.00796-19-st004.docx]

Table S 4. Oligonucleotides used for mutagenesis

For generation of gene deletions

| Designation | Sequence 5´→ 3´ | Template |
| --- | --- | --- |
| *glgA*-Red-Del-for | AGGTCAAACAGGAGCGATAATGCAGGTTTTACATGTATGTATTCCGGGGATCCGTCGACC | pKD13 |
| *glgA*-Red-Del-rev | TAAACGGAGCATTCATATAAATGATTCCTGGATGACTATTTGTAGGCTGGAGCTGCTTCG |  |
| *gltA-*Del13-for | GTACCGGATGGCGAGGGTTGCGTCGCCATCCGGTTGTCAAATTCCGGGGATCCGTCGACC |  |
| *gltA*-Del13-rev | AGGCATTTTGTTGCATCGCGGTTTCCCGATTGACCAGCTTGTAGGCTGGAGCTGCTTCG |  |
| *icdA*-Del13-for | TGACAGACGAGCAAACCAGAAGCGCTCGAAGGAGAGGTGAATTCCGGGGATCCGTCGACC |  |
| *icdA*-Del13-rev | GACGTTAAGTCCCCGTTTTTGTTTTTAACAATTATCGTTAGTAGGCTGGAGCTGCTTCG |  |
| *sucAB*-Del-for | GTATTAAATAAGCAGAAAAGATGCTTAAGGGATCACGATGGTGTAGGCTGGAGCTGCTTC | pKD4 |
| *sucAB*-Del-rev | CCGGCCTACAGGTAGCAGGTGATGCTCTTGCTGACTACACCATATGAATATCCTCCTTAG |  |
| *sucCD*-Del13-for | GGTCTAAAGATAACGATTACCTGAAGGATGGACAGAACACattccggggatccgtcgacc | pKD13 |
| *sucCD*-Del13-rev | GAAAACGGACATTTATCTGTTCCCGCAGGAACAGCGAGTTgtaggctggagctgcttcg |  |
| *mdh*-Del13-for | GCAATAGACACTTAGCTAATCATATAATAAGGAGTTTAGGATTCCGGGGATCCGTCGACC |  |
| *mdh*-Del13-rev | AGAAGCCGGAGCAAAAGCCCCGGCATCGGGCAGGAACAGCGTAGGCTGGAGCTGCTTCG |  |

Oligonucleotides used to confirm gene deletions

| Designation | Sequence 5´→ 3´ |
| --- | --- |
| *glgA*-Del-Check-for | GGTGCAATCTGTGCTCTTCC |
| *glgA*-Del-Check-rev | GCTCCACCAGACGATCGCGC |
| *fumA*-delcheck13-for | CAGAGAATAACCATACCGAG |
| *fumAC*-delcheck13-rev | CAGCAGAAACAGGTGCAAC |
| *fumB*-delcheck13-for | GCTGGATCTTTGCCGCAATG |
| *fumB*-delcheck13-rev | GTGAGCATGGTCTCTCGTG |
| *gltA*-delcheck13-for | GCAGCGATAACAGGAACAAC |
| *gltA*-delcheck13-rev | CTTAAGCAATAAGGCGCTAAG |
| *icdA*-delcheck13-for | CTAACGCAGTCGTGCAGCAG |
| *icdA*-delcheck13-rev | CTATTGCGGTCTGAATTGAG |
| *sucA*-DelCheck-For | CTCGTCATAGTTCACGTTGC |
| *sucB*-DelCheck-Rev | TCTTTAGACCTGTAGGCCTG |
| *sucCD*-DelCheck13-for | TCCTGGTCACCATCAAAGAG |
| *sucCD*-DelCheck13-rev | TAAAGGTGGCCAACCATGTC |
| *mdh*-DelCheck13-for | GCATTCTTGATGAGTGAGG |
| *mdh*-DelCheck13-rev | GGAGTTTAGCAGATTTAGTGC |
| *cheY*-DelCheck-For | CGAAGCAAGTTGTGTGGTG |
| *cheZ*-DelCheck-Rev | AAACCATTCGCGCCGATAG |
| k1-red-del | CAGTCATAGCCGAATAGCCT |
| k2-red-del | CGGTGCCCTGAATGAACTGC |

Oligonucleotides used for generation of complementation and reporter plasmids

| Plasmid | designation | Sequence 5´- 3´ |
| --- | --- | --- |
| p3752 | *fumAC*-komp NotI-for | CGTTAGGCGGCCGCGGCGCACAGTACTTTAAACAG |
|  | *fumAC*-komp XhoI-rev | CCTAGACTCGAGGCATTAATCAACACGGACAAC |
| p3756 | *fumB*-komp ApaI-for | CGTTACGGGCCCTGTGGTAGCGACCAGCGATG |
|  | *fumB*-komp XhoI-rev | CCTAGACTCGAGGTATTATCCCATGCCGAGAGTG |
| p4763 | Vf-pWSK29 | GAATTCCTGCAGCCCGGGG |
|  | Vr-pWSK29 | GGTACCCAATTCGCCCTATAGTGAGTC |
|  | 1f-*glgP*-pWSK29 | CCCGGGCTGCAGGAATTCTACTCGACCCTTTTTCCATGACAGA |
|  | 1r-pWSK29-P*glgB* | TAGGGCGAATTGGGTACCTTGCAGCGCTTATCGGGC |
| p4889 | Vf-p4889 | ATGCGCAAAGGCGAAGAACTGTTTACCGGTGTGGTGCCGA |
|  | Vr-p4889 | GGCCGGCATCACCGGCGCCACAGGTGCGGTTG |
| p5371 | 1f-PwraB-p4889-2 | CCGGTGATGCCGGCCAACCGAAATATTCTGCAACA |
|  | 1r-PwraB-p4889-2 | CTTCGCCTTTGCGCATATTGTACTACTCCTCAGATTAAT |

Oligonucleotides used for qPCR

| *Designation* | Sequence 5‘ – 3‘ | Target gene |
| --- | --- | --- |
| *glgA*-qPCR-for2 | GACCATATAACGGCGGTGAG | *glgA* |
| *glgA*-qPCR-rev2 | CGTTCAGTATGCCGGACAAG |  |
| *glgC*-qPCR-for | CTGGTGACGCCAGCAAATCG | *glgC* |
| *glgC*-qPCR-rev | GGAAAGGATGCGCGTAAGCC |  |
| 16SrRNA-qPCR-for4 | GGTCTGTCAAGTCGGATGTG | 16S rRNA |
| 16SrRNA-qPCR-rev4 | CCTGAGCGTCAGTCTTTGTC |  |
